# Age‐Associated Alterations in Sperm Quality and DNA Integrity in Cryopreserved Bali Bulls (*Bos javanicus*) Semen

**DOI:** 10.1155/vmi/9299197

**Published:** 2026-09-21

**Authors:** Pudji Astuti, Hikmayani Iskandar, Sarmin Sarmin, Claude Mona Airin, Erni Damayanti, Tulus Maulana, Herry Sonjaya

**Affiliations:** ^1^ Department of Physiology, Faculty of Veterinary Medicine, Universitas Gadjah Mada, Yogyakarta, Indonesia, ugm.ac.id; ^2^ Research Center for Applied Zoology, National Research and Innovation Agency (BRIN), Bogor, Indonesia, brin.go.id; ^3^ Department of Animal Production, Faculty of Animal Science, Universitas Hasanuddin, Makassar, Indonesia, unhas.ac.id

**Keywords:** *Bos javanicus*, cryopreservation, DNA integrity, spermatozoa, testosterone

## Abstract

Age‐related reproductive changes in breeding bulls may influence semen quality and long‐term efficiency of artificial insemination (AI) programs. However, molecular indicators of aging, particularly in indigenous tropical cattle such as Bali cattle (*Bos javanicus*), remain insufficiently characterized. This study aimed to evaluate age‐associated alterations in post‐thaw sperm kinematics, DNA integrity, protamine integrity, and testosterone concentrations in Bali bulls. Frozen semen from 10 bulls was categorized into two age groups (< 8 years and > 10 years; *n* = 5 per group). Sperm motility parameters were evaluated using computer‐assisted sperm analysis (CASA), DNA integrity was assessed by sperm‐bos‐halomax, protamine integrity using chromomycin A3 staining, and testosterone concentrations were measured using ELISA. Total and progressive motility did not differ significantly between age < 8 years and > 10 years (*p* > 0.05). However, bulls > 10 years exhibited significantly higher VSL, DCL, and BCF values, while sperm DNA integrity was significantly reduced. Notably, DNA integrity was significantly reduced in older bulls (91.97 ± 1.32%) compared with younger bulls (97.82 ± 0.48%; *p* = 0.01), whereas protamine integrity and testosterone concentrations showed no significant age‐related differences. Correlation analysis revealed a strong positive association between progressive motility and DNA integrity in younger bulls (*r* = 0.84), whereas in older bulls, progressive motility was strongly negatively correlated with testosterone concentration (*r* = −0.91). These findings indicate that reproductive aging in Bali bulls is primarily reflected by impaired genomic integrity rather than alterations in conventional semen quality or endocrine status. Although total motility, progressive motility, protamine integrity, and testosterone concentrations did not differ significantly between bulls aged < 8 years and > 10 years, sperm DNA integrity was significantly lower in bulls aged > 10 years than in bulls aged < 8 years. These findings suggest that sperm DNA integrity may serve as a more sensitive biomarker of reproductive aging than conventional semen characteristics and may improve the accuracy of breeding soundness evaluation in AI programs.

## 1. Introduction

Bali cattle (*Bos javanicus*) represent one of Indonesia’s most important indigenous genetic resources and contribute substantially to national beef production through artificial insemination (AI) programs [1]. The prolonged use of genetically superior bulls in AI centers, often beyond 10 years of age, necessitates a comprehensive understanding of age‐related reproductive changes. Although mature bulls may retain acceptable semen production and field fertility, advancing age has been associated with subtle alterations in sperm functional competence and molecular integrity in several livestock species [2, 3]. Given the strategic importance of Bali cattle in tropical production systems, evaluating the impact of aging on semen quality is essential to ensure sustained reproductive efficiency and genetic progress.

Aging in domestic animals is characterized by progressive modifications in spermatogenesis, including increased oxidative stress, mitochondrial dysfunction, and cumulative DNA damage. In bovine species, conventional semen parameters such as motility and concentration do not always decline markedly with age, particularly in well‐managed breeding bulls [3, 4]. Besides sperm DNA integrity, reproductive aging may affect multiple physiological processes involved in male fertility [5]. Testosterone is a key regulator of spermatogenesis, epididymal sperm maturation, and maintenance of normal reproductive function. Age‐related changes in androgen production may influence semen quality [6]. Moreover, computer‐assisted sperm analysis enables objective, quantitative evaluation of sperm motion using velocity‐ and trajectory‐based kinematic parameters that have been linked to fertilizing potential beyond conventional motility assessment. At the molecular scale, changes in the expression and composition of proteins of sperm and seminal plasma have been associated with oxidative stress, chromatin remodeling, and impaired sperm function during aging. Thus, combining endocrine, kinematic, and molecular biomarkers with conventional semen evaluation may offer a more comprehensive evaluation of reproductive aging and fertility potential in breeding bulls [5, 6]. Elevated DNA fragmentation has been linked to reduced fertilization success, impaired embryo development, and lower pregnancy rates in cattle, underscoring the importance of genomic stability in breeding soundness evaluation [7].

Cryopreservation, a cornerstone of AI programs, imposes additional stress on spermatozoa through osmotic shifts, membrane phase transitions, and reactive oxygen species (ROS) generation. These processes can exacerbate pre‐existing age‐related vulnerabilities at the molecular level, potentially affecting chromatin integrity and post‐thaw functionality [8, 9]. While post‐thaw motility is routinely assessed in AI centers, fewer studies have simultaneously examined DNA integrity, chromatin packaging, and endocrine status in aging bulls, particularly in *B*. *javanicus*. Moreover, the relationship between testosterone concentrations and post‐thaw sperm quality remains incompletely understood, as systemic androgen levels may remain stable despite cellular or genomic alterations within the testes [10, 11].

Therefore, the present study aimed to investigate age‐associated alterations in post‐thaw sperm quality, DNA integrity, protamine integrity, and circulating testosterone concentrations in Bali bulls. By integrating functional (motility), molecular (DNA and chromatin integrity), and endocrine parameters, this study aimed to determine whether reproductive aging in *B. javanicus* is primarily reflected in conventional semen characteristics or at the genomic level. Elucidating these relationships will contribute to evidence‐based breeding management strategies and enhance the precision of reproductive evaluation protocols in tropical indigenous cattle populations.

## 2. Materials and Methods

### 2.1. Experimental Design and Bull Determination

The study was conducted between November 2025 and February 2026. Frozen semen and blood samples were obtained from the Regional Artificial Insemination Center (RAIC), where Bali bulls are maintained under standardized management conditions. The frozen semen used in this study was obtained as commercial cryopreserved semen produced by the RAIC. All ejaculates had been collected using an artificial vagina within the same collection period to minimize seasonal variation and were cryopreserved using the same extender (AndroMed, Minitüb, Tiefenbach, Germany). To minimize potential variability among animals, all bulls were maintained under identical environmental conditions, feeding regimes, and handling practices. The bulls were housed individually in 2.5 × 2.0 m pens equipped with feeding and drinking facilities. They were fed fresh forage and a commercial concentrate twice daily (morning and afternoon) according to the standard feeding protocol of the RAIC to meet their nutritional requirements, and fresh drinking water was provided ad libitum throughout the study period. All procedures implemented at the AI center complied with Indonesian national operational standards (SNI ISO 9001:2015 No. 824 100 16072) and were conducted under the supervision of licensed veterinarians and adhered to animal welfare principles in accordance with the Animal Care and Use Committee’s ethical guidelines. The bulls included in this study were owned and managed by the government‐operated RAIC. A total of 100 straws of cryopreserved semen (10 straws per bull) from 10 Bali bulls were used to assess sperm motility, DNA integrity, protamine integrity, and scanning electron microscopy (SEM). The bulls were classified into two age groups according to their chronological age: a younger group (6–8 years, *n* = 5) and an older group (10–12 years, *n* = 5). These age groups were selected to facilitate comparisons between relatively younger and older sexually mature Bali bulls and to maximize the contrast in age‐related reproductive characteristics.

### 2.2. Frozen‐Thawed Semen

Only ejaculates meeting the minimum freezing criteria (progressive motility ≥ 70%) were subjected to cryopreservation according to the standardized protocol implemented at the RAIC, as previously described in [6]. After storage in liquid nitrogen (−196°C), individual semen straws were thawed in a 37°C water bath for 30 s. Ten frozen semen straws from each bull were evaluated in this study. The thawed semen was transferred into sterile microcentrifuge tubes and assessed for post‐thaw quality. Only ejaculates exhibiting a post‐thaw total motility of ≥ 40%, normal sperm morphology, and no visible evidence of contamination or physical abnormalities were included in the subsequent analyses. To avoid potential alterations in sperm quality resulting from repeated handling, each thawed semen straw was used for a single analytical procedure. Separate semen straws from the same bull were allocated for computer‐assisted sperm analysis (CASA), sperm DNA integrity assessment, chromomycin A3 (CMA3) staining, and SEM. All analyses were performed immediately after thawing under identical laboratory conditions.

### 2.3. Sperm Evaluation by CASA

Sperm motility was assessed by placing a 10‐μL aliquot of semen on a prewarmed glass slide and covering it with a coverslip. Motility analysis was performed using a computer‐assisted sperm analysis system (CASA; Sperm Vision 3.7, Minitüb, Tiefenbach, Germany). The evaluated parameters included total motility (%), progressive motility (%), distance average path (DAP, µm/s), distance curved line (DCL, µm/s), distance straight line (DSL, µm/s), average path velocity (VAP, µm/s), velocity curve linear (VCL, µm/s), velocity straight length (VSL, µm/s), straightness (STR, %), linearity (LIN, %), wobble (WOB, %), amplitude of lateral head displacement (ALH, µm), and beat cross frequency (BCF, Hz), defined as the frequency at which the sperm head crosses its average path. For each semen sample, a minimum of 200 spermatozoa from 10 randomly selected microscopic fields were analyzed under a light microscope at 200x magnification.

### 2.4. Protamine Integrity

**FIGURE 1:**
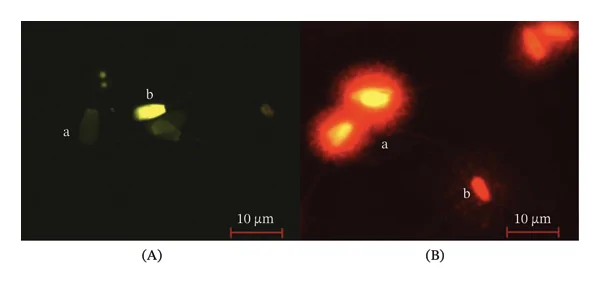
Magnification 400x, microscope fluorescence, imager Z2, Carl Zeiss, Germany. (A) Spermatozoa protamine integrity: (a) protamine deficiency, (b) protamine integrity; (B) DNA integrity: (a) DNA intact (large halo), (b) DNA fragmented (small or bull halo).

Protamine integrity was evaluated using CMA3 staining. Sperm suspensions were washed twice in phosphate‐buffered saline (PBS), fixed in ethanol:chloroform:acetic acid (6:3:1, v/v/v) for 8 min at 4°C, and allowed to air‐dry. Subsequently, slides were treated with 100 μL of CMA3 solution (0.25 mg/mL in McIlvaine buffer supplemented with 10 mM MgCl_2_, pH 7.0; Sigma‐Aldrich, USA) and incubated for 30 min at 4°C. Following incubation, the slides were gently rinsed with McIlvaine buffer, dried, and mounted with an antifade medium (Fluoprep, bioMérieux, France). Fluorescence assessment was performed using a fluorescence microscope (AxioVision) equipped with an excitation wavelength of 460–470 nm. Bright yellow fluorescence (CMA3‐positive) indicated protamine deficiency, whereas weak or dull fluorescence (CMA3‐negative) indicated normal protamine content (Figure 1A).

### 2.5. DNA Integrity

DNA integrity was evaluated using a modified sperm chromatin dispersion (SCD) assay with the Sperm–Bos–Halomax® kit (Halotech DNA, SL, Spain), in accordance with the procedures described by Said et al. [12]. A total of 200 spermatozoa per slide were examined. Spermatozoa exhibiting a clearly defined halo surrounding the head were classified as having fragmented DNA, whereas those without halo formation were considered to possess nonfragmented DNA (Figure 1B).

### 2.6. Measurement of Testosterone

Blood samples were collected in the morning by trained veterinarians and technical staff at the RAIC as part of the routine bull management program. To minimize physiological variation, all blood samples were collected under standardized management conditions using the same sampling procedure for all bulls. Approximately 3–5 mL of blood was collected from the jugular vein of each bull into 5‐mL EDTA‐coated vacutainer tubes (Terumo, USA). Samples were centrifuged at 3000 rpm for 10 min at room temperature to separate the plasma. The harvested plasma was transferred into microtubes and stored at −20°C until hormonal analysis. Testosterone concentrations were quantified using a bovine testosterone enzyme‐linked immunosorbent assay kit (ELISA; Signalway Antibody, #EK0019) according to the manufacturer’s instructions. Prior to analysis, plasma samples were diluted 1:4 with distilled water. Standard solutions ranging from 0.2 to 16 ng/mL were prepared, and 25 μL of standards and diluted samples were loaded into ELISA plate wells in duplicate. Enzyme conjugate was added to all wells except blanks, and the plate was gently mixed and incubated at room temperature for 60 min. Following incubation, the wells were washed three to four times with 300 μL of washing buffer per cycle. Subsequently, 200 μL of substrate solution was added and incubated for 15 min at room temperature. The enzymatic reaction was terminated by adding 100 μL of stop solution to each well. Absorbance was measured at 450 nm using a microplate reader.

### 2.7. Preparation for SEM

Frozen semen straws were thawed at 37°C for 30 s. The semen samples were washed twice with PBS by centrifugation at 3000 rpm for 10 min to remove the extender, and the pellet was resuspended to a final volume of approximately 1 mL. The washed sperm pellet was subsequently rinsed with ultrapure water and resuspended in 500 μL of ultrapure water prior to SEM specimen preparation and imaging.

### 2.8. Field Emission Scanning Electron Microscopy (FESEM)

**FIGURE 2:**
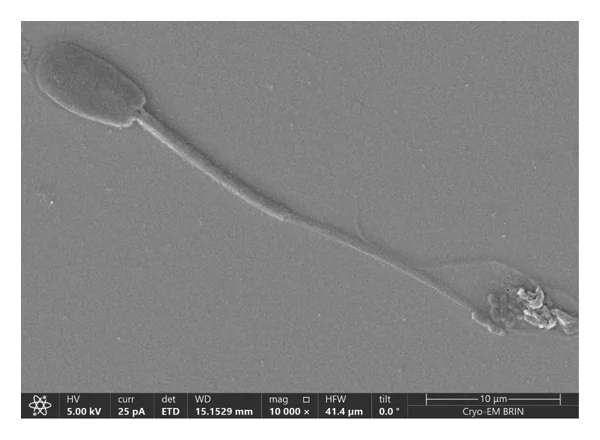
Morphology of spermatozoa after post‐thawing of Bali bulls using scanning electron microscopy (SEM) at 3500x magnification.

FESEM was conducted using a Dual‐Beam FIB Aquilos 2 system (Thermo Fisher Scientific, USA), equipped with a field emission gun (FEG) and operated under low‐temperature conditions (< 0°C). Imaging was performed at an accelerating voltage of 5 kV and magnifications up to 25000x to examine the surface morphology of Bali bull semen. Samples were mounted on SEM stubs and subsequently transferred under high‐vacuum conditions from the preparation station to the chamber of the Aquilos2 system. The microscope stage allows 360° rotation and multiangle tilting, providing optimal flexibility for imaging from diverse orientations. To enhance surface conductivity, a thin platinum layer (∼5 nm) was deposited onto the samples for 15 s at a current of 30 mA using an integrated retractable sputter coater. These parameters ensured the acquisition of high‐resolution images without the need to remove the samples during the observation process (Figure 2).

### 2.9. Statistical Analysis

The data were analyzed statistically using SPSS ver. 25.0 (IBM Corp., Armonk, NY, USA). Data were presented as the mean ± standard deviation (SD) and differences were considered statistically significant at *p* < 0.05. Prior to statistical analysis, data normality was evaluated using the Shapiro–Wilk test, and homogeneity of variance was assessed using Levene’s test. An independent *t*‐test was performed to compare the mean values of CASA parameters between age groups. Pearson’s correlation test was applied to evaluate the relationships among progressive motility, DNA integrity, protamine deficiency, and testosterone levels. The correlation matrix was subsequently visualized as a heatmap using RStudio.

## 3. Results

### 3.1. Post‐Thaw Sperm Motility and Kinematic Parameters

Computer‐assisted sperm analysis demonstrated that age influenced several motion characteristics of frozen–thawed sperm obtained from Bali bulls (Table 1). Although total motility was comparable between age groups (< 8 years: 52.20 ± 3.90% vs. > 10 years: 55.72 ± 4.49%; *p* = 0.87), specific velocity‐related parameters differed. Sperm from the > 10‐year group showed greater DCL values than those from the < 8‐year group (45.80 ± 4.65 vs. 40.68 ± 2.41 μm/s; *p* = 0.02). A similar pattern was observed for straight‐line velocity, which was higher in the > 10‐year group (54.79 ± 10.68 μm/s) compared with the < 8‐year group (45.54 ± 4.58 μm/s; *p* = 0.01). BCF was also significantly increased in the > 10‐year group (25.75 ± 4.30 vs. 23.27 ± 2.13 Hz; *p* = 0.02). Curvilinear velocity tended to be higher in bulls aged > 10 years (110.43 ± 17.78 vs. 94.45 ± 8.47 μm/s), approaching the threshold of statistical significance (*p* = 0.05). No differences were observed in other CASA parameters, including DAP (28.98 ± 3.87 vs. 37.08 ± 6.03 μm/s; *p* = 0.34), DSL (21.50 ± 3.46 vs. 27.59 ± 6.24 μm/s; *p* = 0.28), or VAP (65.74 ± 4.51 vs. 75.24 ± 9.63 μm/s; *p* = 0.11). Likewise, trajectory‐related parameters, including STR, LIN, WOB, and ALH, remained comparable between the < 8‐year and > 10‐year groups (*p* > 0.05).

**TABLE 1:** CASA‐derived sperm kinematic parameters of cryopreserved semen from Bali bulls of different ages.

Parameters	Age group		Sig. (0.05)
	< 8 years (*n* = 5)	> 10 years (*n* = 5)	
Total motility (%)	52.20 ± 3.90	55.72 ± 4.49	0.87
DAP (μm/s)	28.98 ± 3.87	37.08 ± 6.03	0.34
DCL (μm/s)	40.68 ± 2.41	45.80 ± 4.65	0.02
DSL (μm/s)	21.50 ± 3.46	27.59 ± 6.24	0.28
VAP (μm/s)	65.74 ± 4.51	75.24 ± 9.63	0.11
VCL (μm/s)	94.45 ± 8.47	110.43 ± 17.78	0.05
VSL (μm/s)	45.54 ± 4.58	54.79 ± 10.68	0.01
STR (%)	68.40 ± 5.12	72.00 ± 6.16	0.52
LIN (%)	52.13 ± 3.29	51.73 ± 3.19	0.87
WOB (%)	71.93 ± 1.19	70.14 ± 2.53	0.28
ALH (μm)	5.33 ± 0.53	5.39 ± 0.43	0.52
BCF (Hz)	23.27 ± 2.13	25.75 ± 4.3	0.02

*Note:* Values are presented as mean ± standard deviation (SD); *n* = number of bulls per group. Sig. (0.05) indicates the *p* value from independent *t*‐test analysis; values of *p* < 0.05 are considered statistically significant.

### 3.2. Sperm DNA Integrity and Protamine Status

Post‐thaw sperm quality assessment revealed that sperm DNA integrity was the only parameter that differed significantly between age groups (Table 2). Bulls < 8‐year group exhibited a higher proportion of spermatozoa with intact DNA compared with bulls older than 10 years (97.82 ± 0.48% vs. 91.97 ± 1.32%, respectively; *p* = 0.01). In contrast, progressive motility remained comparable between the two age categories. The mean percentage of progressively motile spermatozoa was 37.02 ± 5.32% in the < 8‐year group and 46.32 ± 4.67% in the > 10‐year group, with the observed numerical difference not reaching statistical significance (*p* = 0.70). Similarly, protamine integrity did not show a significant age‐related variation. The proportion of sperm cells exhibiting normal chromatin packaging was 95.97 ± 1.02% in bulls aged < 8 years and 93.76 ± 2.69% in those aged > 10 years (*p* = 0.23). The distribution of values indicated substantial overlap between groups, suggesting relative stability of protamine status despite advancing age. These findings indicate that, although sperm motility and chromatin packaging were maintained, sperm DNA integrity declined in bulls aged > 10 years.

**TABLE 2:** Age‐dependent variation in progressive motility, DNA integrity, and protamine integrity.

Parameters	Age group		Sig. (0.05)
	< 8 years (*n* = 5)	> 10 years (*n* = 5)	
Progressive motility (%)	37.02 ± 5.32	46.32 ± 4.67	0.70
DNA integrity (%)	97.82 ± 0.48	91.97 ± 1.32	0.01
Protamine integrity (%)	95.97 ± 1.02	93.76 ± 2.69	0.23

*Note:* Values are presented as mean ± standard deviation (SD). *N* = 5 bulls per group. Sig. (0.05) indicates *p* values from independent sample *t*‐test; values of *p* < 0.05 are considered statistically significant.

### 3.3. Testosterone Concentration

Testosterone concentrations are presented in Table 3. Bulls aged < 8 years had a mean testosterone concentration of 33.31 ± 22.48 ng/mL, whereas bulls aged > 10 years exhibited a mean concentration of 36.00 ± 27.29 ng/mL. No significant difference was detected between the two age groups (*p* = 0.65). Substantial interindividual variation was observed in both groups, as reflected by the relatively large SDs and overlapping distributions. Overall, these results indicate that testosterone concentrations were not significantly affected by age in the evaluated Bali bulls.

**TABLE 3:** Testosterone concentration in different‐age Bali bulls.

Parameters	Age group		Sig. (0.05)
	< 8 years (*n* = 5)	> 10 years (*n* = 5)	
Testosterone (ng/mL)	33.31 ± 22.48	36.00 ± 27.29	0.65

*Note:* Data are presented as mean ± standard deviation (SD); *n* = 5 bulls per group. Sig. (0.05) represents the *p* value from an independent *t*‐test analysis. A *p* > 0.05 indicates no statistically significant difference between age groups.

### 3.4. Correlation Analysis Between Semen Quality Parameters and Testosterone Concentration

**FIGURE 3:**
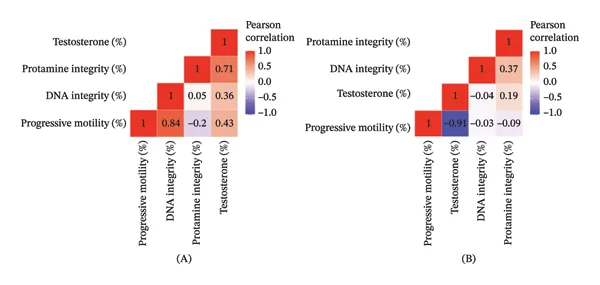
Pearson correlation heatmap analysis of progressive motility, DNA integrity, protamine integrity, and testosterone levels in Bali bulls. (A) Bulls aged < 8 years; (B) bulls aged > 10 years. The color scale indicates the Pearson correlation coefficient (*r*), ranging from blue (strong negative correlation, −1.0) to red (strong positive correlation, +1.0).

In bulls aged < 8 years (Figure 3A), progressive sperm motility exhibited a very strong positive correlation with DNA integrity (*r* = 0.84, *p* = 0.075), indicating that improved sperm motility tended to be associated with better DNA integrity, although the relationship did not reach statistical significance. In contrast, in bulls aged > 10 years (Figure 3B), progressive sperm motility showed a very strong negative correlation with testosterone concentration (*r* = −0.91, *p* = 0.031), indicating a statistically significant inverse relationship. The heatmap color scale provides a visual representation of the strength and direction of these correlations, with dark red indicating strong positive correlations and dark blue indicating strong negative correlations. Lighter colors or white indicate weak or no correlation among the variables examined. Correlation mapping further demonstrated that relationships among functional and molecular parameters differed between age categories. In the < 8‐year group, progressive motility was strongly and positively associated with DNA integrity (*r* = 0.84). Conversely, in the > 10‐year group, progressive motility displayed a strong negative correlation with testosterone concentration (*r* = −0.91), while other associations were weak or absent.

## 4. Discussion

Advancing age selectively influenced post‐thaw sperm kinematic characteristics in Bali bulls, whereas total motility remained statistically comparable between age groups. The preservation of total motility suggests that chronological aging does not necessarily impair the proportion of motile spermatozoa surviving cryopreservation; however, velocity‐related parameters appeared more sensitive to age‐associated modulation. Bulls older than 10 years exhibited significantly higher VSL, DCL, and BCF, with VCL approaching statistical significance. Contemporary studies have emphasized that CASA‐derived velocity metrics, particularly VSL and VCL, are more predictive of fertilization competence than total motility alone [5, 13]. In bovine spermatozoa, such parameters reflect mitochondrial ATP production efficiency and the integrity of axonemal microtubule function, both of which may undergo age‐related metabolic reprogramming [14, 15]. Therefore, the elevated velocity observed in older bulls may represent adaptive metabolic adjustments rather than a straightforward decline in functional capacity.

The increase in BCF in older bulls further supports the hypothesis of altered flagellar regulation with advancing age. BCF is associated with oscillatory flagellar activity and is influenced by intracellular calcium flux, membrane lipid composition, and ATP availability [16]. Age‐dependent changes in sperm membrane lipid remodeling and oxidative balance have been reported in several livestock species, potentially affecting cryo‐resilience and post‐thaw motility kinetics [3, 17]. Notably, STR, LIN, WOB, and ALH remained stable between age groups. This preservation of movement trajectory suggests that although kinetic intensity (velocity magnitude) was modified, the coordination of flagellar beating and directional efficiency were maintained. Such findings indicate that aging may influence the energetic output of spermatozoa without fundamentally disrupting axonemal structural integrity.

Collectively, these data indicate that aging in Bali bulls does not uniformly deteriorate post‐thaw sperm motility but rather induces selective alterations in velocity‐related kinematic attributes. This pattern aligns with recent evidence suggesting that sperm aging is characterized more by qualitative bioenergetic remodeling than by abrupt functional collapse [18]. Given that specific CASA velocity parameters have been positively correlated with in vivo fertility outcomes in cattle [19], the observed age‐associated kinetic modulation may have practical implications for semen selection in AI programs.

DNA integrity was the only post‐thaw sperm quality parameter that differed significantly between age groups, with younger Bali bulls (< 8 years) exhibiting a markedly higher proportion of spermatozoa with intact DNA compared with bulls older than 10 years. This finding indicates that advancing age may preferentially compromise genomic stability rather than gross motility traits. The reduction of approximately 6% in DNA‐intact spermatozoa in the older group is biologically relevant, considering that even moderate increases in DNA fragmentation have been associated with reduced embryo development and pregnancy rates in cattle [20, 21]. Similar age‐associated declines in sperm DNA integrity have been reported in bovine and other domestic species, where oxidative stress accumulation and diminished DNA repair capacity in germ cells were proposed as underlying mechanisms [3, 22]. Cryopreservation itself is known to exacerbate oxidative damage and chromatin destabilization, potentially amplifying subtle age‐related vulnerabilities [23, 24]. Therefore, the lower DNA integrity observed in bulls > 10 years may reflect an interaction between physiological aging and cryo‐induced oxidative stress.

In contrast, progressive motility did not differ significantly between age categories, despite a numerically higher mean value in the older group. This observation aligns with reports indicating that conventional motility parameters are less sensitive indicators of reproductive aging than molecular markers such as DNA fragmentation or chromatin condensation status [25, 26]. Several studies in bovine semen have demonstrated that bulls with comparable progressive motility may differ substantially in fertilizing capacity due to differences in DNA integrity or mitochondrial functionality [27, 28]. Thus, the maintenance of progressive motility in older Bali bulls does not necessarily imply preserved genomic competence. Instead, it suggests that functional movement and nuclear integrity may deteriorate at different rates during aging, supporting the concept of asynchronous reproductive senescence.

Interestingly, protamine integrity and chromatin packaging remained statistically stable across age groups, with considerable overlap in distribution. This finding suggests that large‐scale protamination defects were not the primary driver of increased DNA damage in older bulls. Previous studies reported that DNA fragmentation can increase independently of measurable alterations in protamine content, particularly when damage arises from oxidative strand breaks rather than defective chromatin remodeling during spermatogenesis [22, 29, 30]. In bovine species, age‐related increases in ROS production have been associated with higher DNA fragmentation indices without concurrent changes in protamine ratios [29, 31]. Therefore, the present study indicates that while chromatin packaging remains relatively preserved, DNA strand integrity becomes more susceptible with advancing age. Collectively, these findings suggest that genomic stability may serve as a more sensitive biomarker of reproductive aging in *B. javanicus* than conventional motility or protamine‐based parameters, underscoring the importance of integrating DNA integrity assessment into routine semen quality evaluation for AI programs.

Testosterone concentrations did not differ significantly between Bali bulls aged < 8 years and those > 10 years, despite the numerically higher mean value observed in the older group. The substantial intragroup variability and overlapping distributions indicate that chronological age beyond 10 years was not associated with a consistent endocrine shift under the present management and sampling conditions. In bovine species, testosterone secretion is characterized by pulsatile release and marked individual variation influenced by nutritional status, environmental factors, and sampling time, which may obscure subtle age‐related trends [32]. Several recent studies in cattle have similarly reported stable testosterone concentrations in mature and late‐mature bulls maintained under optimal health and management, suggesting that Leydig cell steroidogenic capacity can remain functionally preserved despite advancing age [33, 34]. Therefore, the absence of endocrine decline in the present study aligns with growing evidence that reproductive aging in bulls does not necessarily manifest as a marked reduction in systemic androgen levels.

Importantly, the stability of testosterone concentrations contrasts with the observed reduction in sperm DNA integrity in older bulls, indicating that genomic deterioration may occur independently of detectable hormonal insufficiency. Previous studies reported that increased sperm DNA fragmentation in aging males is more closely associated with oxidative stress accumulation, mitochondrial dysfunction, and impaired chromatin stabilization rather than diminished circulating testosterone [31, 35]. In bovine semen, cryopreservation can further exacerbate oxidative damage, potentially amplifying latent age‐related vulnerabilities at the molecular level. The dissociation between preserved endocrine status and reduced DNA integrity observed here supports the concept that reproductive senescence in bulls may initially manifest at the genomic and cellular levels before endocrine alterations become evident. Collectively, these findings suggest that testosterone alone may not serve as a sensitive biomarker of reproductive aging in Bali bulls and underscore the importance of integrating molecular sperm quality assessments into breeding soundness evaluations.

Correlation mapping revealed age‐dependent differences in the interaction between functional and molecular sperm parameters. In bulls younger than 8 years, progressive motility was strongly and positively correlated with DNA integrity, indicating a close coupling between functional competence and genomic stability in early mature animals. This relationship supports the concept that, in younger bulls, sperm movement efficiency reflects underlying chromatin integrity and overall cellular health. In contrast, in bulls older than 10 years, progressive motility exhibited a strong negative correlation with circulating testosterone concentrations, while associations with DNA integrity and other parameters were weak or absent. This shift in correlation patterns suggests a decoupling between motility and genomic stability with advancing age and may reflect altered endocrine–sperm functional interactions in older animals. The emergence of a negative association between testosterone and motility in the older group could indicate compensatory endocrine responses, altered androgen receptor sensitivity, or dysregulated spermatogenic signaling rather than a direct stimulatory effect of testosterone on post‐thaw sperm function. Collectively, these findings highlight that aging in Bali bulls is accompanied not only by changes in individual sperm quality parameters but also by a reorganization of the biological relationships linking endocrine, functional, and molecular determinants of semen quality.

A limitation of the present study is the relatively small sample size, with five bulls included in each age group. This sample represented the entire population of Bali bulls available at the AI center during the study period, thereby limiting the possibility of increasing the number of experimental animals. Although statistically significant differences were detected for several parameters, the limited sample size may have reduced the statistical power to detect more subtle age‐related effects. Likewise, the correlation analyses should be interpreted with caution because correlation coefficients derived from a small number of observations may be sensitive to individual variation. Therefore, further studies involving larger populations from multiple AI centers are warranted to validate and extend the present findings.

## 5. Conclusion

Aging in Bali cattle primarily reduces sperm DNA integrity, while motility and testosterone levels remain stable. This highlights the importance of assessing sperm DNA in routine semen evaluation to improve bull selection accuracy.

## Funding

This work was supported by Riset Kolaborasi Indonesia (RKI) EQUITY, 13609/UN1.P2/Dit‐Lit/PT.01.03/2025.

## Disclosure

All authors have read and approved the final version of the manuscript.

## Ethics Statement

Pudji Astuti affirms that this manuscript is an honest, accurate, and transparent account of the study being reported; that no important aspects of the study have been omitted; and that any discrepancies from the study as planned (and, if relevant, registered) have been explained.

## Conflicts of Interest

The authors declare no conflicts of interest.

## Data Availability

The data that support the findings of this study are available from the corresponding author upon reasonable request.
